# If You Don’t Use It, You Lose It: Perspectives of Older Adults on Aging in Place in Subsidized Housing

**DOI:** 10.1093/geroni/igaa057.644

**Published:** 2020-12-16

**Authors:** David Reyes-Farias, Momana Jahan, Sonia Pandit, Carolina Reid, Barbara Resnick, Rebecca Brown

**Affiliations:** 1 University of Pennsylvania, Philadelphia, Pennsylvania, United States; 2 University of California, Berkeley, Berkeley, California, United States; 3 University of Maryland School of Nursing, Baltimore, Maryland, United States

## Abstract

Nearly 3 million older Americans with low incomes live in subsidized housing. This population has disproportionate rates of functional impairment, cognitive impairment, and nursing home admission. Patient-centered interventions are needed to improve aging in place for this population, but little is known about resident perspectives on this topic. We interviewed 58 residents aged 62 or older and 8 caregivers from 7 housing sites. We analyzed transcripts using qualitative thematic analysis. Participants reported that several factors impacted their ability to age in place. First, most participants noted the importance of physical environment, including the design and location of their apartment building. Physical accessibility and proximity to community resources facilitated aging in place, while features such as heavy doors and smoke in communal areas posed challenges. Second, most participants identified the importance of the building’s social environment; support received from other residents and on-site staff facilitated aging in place, while gossip and unpleasant residents were barriers. Third, participants noted that health issues such as arthritis limited their ability to function independently, regardless of environment. Participants emphasized the importance of physical activity for preventing functional decline, stating, “if you don’t use it, you lose it.” Older adults living in subsidized housing view their environment as central to their ability to age in place. Our findings suggest that interventions to improve aging in place in these settings must focus not only on optimizing residents’ physical function, but also on using the environment to promote resident independence and quality of life.

